# Cellular Senescence in the Ascending Aorta and Complexity of Coronary Atherosclerosis

**DOI:** 10.1155/jare/9142131

**Published:** 2026-01-29

**Authors:** Hanife Abanus, Mutlu Vural, Fahrettin Katkat, Esra Paşaoğlu, Abdullah Olgun, Bülent Mert

**Affiliations:** ^1^ Cardiology Department, Medical Sciences University Bağcılar Training and Research Hospital, Istanbul, Turkey; ^2^ Cardiology Department, Medical Sciences University İstanbul Training and Research Hospital, Istanbul, Turkey; ^3^ Pathology Department, Medical Sciences University Bağcılar Training and Research Hospital, Istanbul, Turkey; ^4^ Faculty of Pharmacy, Biochemistry Department, İstinye University, Istanbul, Turkey; ^5^ Cardiovascular Surgery Department, Medical Sciences University Bağcılar Training and Research Hospital, Istanbul, Turkey

**Keywords:** biomarkers, cellular senescence, coronary angiography, coronary artery disease, scoring methods

## Abstract

**Background:**

Cellular senescence might have a key role in the pathogenesis of vascular aging and atherosclerosis. However, human data directly linking cellular senescence in the vascular tissue with coronary artery disease (CAD) are limited. This study aimed to investigate the relationship between the proportion of senescent cells in the ascending aorta and the complexity of CAD in patients undergoing coronary artery bypass grafting (CABG).

**Methods:**

Ascending aortic tissue samples were obtained from 112 patients during elective or urgent CABG surgery. Expressions of p16, p21, and β‐galactosidase (β‐gal) were evaluated as cellular senescence biomarkers using immunohistochemical analysis. The complexity of coronary lesions was quantified by the Synergy between PCI with Taxus and Cardiac Surgery (SYNTAX) score. Patients were stratified into low and moderate‐to‐high SYNTAX score groups, and biomarker expression levels were compared between these subgroups.

**Results:**

The proportion of p16‐positive cells in the ascending aorta was significantly higher in patients with moderate‐to‐high SYNTAX scores, by both percentage (*p* = 0.015) and staining grade (*p* = 0.035). Similarly, p21 expression was elevated in the moderate‐to‐high SYNTAX group (percentage, *p* = 0.015; grade, *p* = 0.030). β‐gal expression showed no significant association with CAD complexity. In multivariate logistic regression analysis, p16 expression remained an independent predictor of higher SYNTAX score (OR: 1.016; 95% CI: 1.000–1.031; *p* = 0.047).

**Conclusion:**

Increased expression of p16 and p21 in ascending aortic tissue is significantly associated with higher coronary atherosclerosis complexity in patients undergoing CABG. Among these biomarkers, p16 serves as an independent predictor of complex CAD, highlighting its potential role in vascular aging and atherosclerosis progression.

## 1. Introduction

Age, gender, family history of premature atherosclerosis, smoking, hypertension, dyslipidemia, diabetes, obesity, sedentary lifestyle, and psychosocial factors are included among risk factors for coronary atherosclerosis. Among these, age is considered one of the most important risk factors for coronary atherosclerosis in risk estimation scales, such as the Framingham Risk Score [1–3]. However, increasing evidence suggests that biological age rather than chronological age provides a more accurate reflection of cardiovascular risk [4–6]. Cellular senescence should be associated with biological age rather than chronological age, as previous studies have shown that cellular senescence may not usually be associated with chronological age [7, 8].

Both animal and human studies have shown that senescent cells accumulate in many tissues with age, including the heart and arteries [9–12]. It has been suggested that this accumulation of senescent cells contributes to the pathogenesis of cardiovascular diseases. Senescence‐associated cellular biomarkers were used to indicate cellular senescence in cardiovascular tissues. To avoid false‐positive findings when cellular senescence biomarkers are used alone, a combination of markers, including high expression of the cyclin‐dependent kinase inhibitors p16 and p21, as well as the activity of the lysosomal hydrolase senescence‐associated β‐galactosidase (β‐gal), has been recommended and evaluated in several studies [13–16].

The Synergy between PCI with Taxus and Cardiac Surgery (SYNTAX) score is recommended to determine the most appropriate revascularization strategy in patients with critical coronary artery stenoses and predict major adverse events after myocardial revascularization in both European and American revascularization guidelines [17–19]. The SYNTAX score (SS) had been used to estimate the complexity of the coronary artery disease (CAD) in our patients who planned to have elective coronary artery bypass grafting (CABG) surgery.

To our knowledge, this is the first study designed to investigate the association between the complexity of CAD, as determined by the SYNTAX score, and the expression of cellular senescence biomarkers (p16, p21, and β‐gal) in vascular tissue obtained from ascending aortic tissue of patients undergoing CABG.

## 2. Material and Methods

A total of 112 consecutive patients who underwent elective or urgent CABG at the Health Sciences University Bağcılar Training and Research Hospital between September 2021 and June 2022 were included in this study after approval by our institutional ethical board (Bakirkoy; approval date: 06.09.2021 approval number: 2021‐17‐21). Detailed anamnesis, physical examination, and biochemical and echocardiographic analyses were performed before CABG surgery. Preoperative and postoperative electrocardiography samples were also recorded. Echocardiographic examination was performed using the iE33 xMATRIX Echocardiography System (Philips, Eindhoven, The Netherlands). Measurement of heart chambers, end‐diastolic and end‐systolic diameters, and evaluation of heart valve functions were routinely performed according to current guidelines [20]. Left ventricular ejection fraction was measured using the modified Simpson method.

### 2.1. Inclusion and Exclusion Criteria for the Participants

#### 2.1.1. Inclusion Criteria

• Age ≥ 18 years
• Indication for elective or urgent CABG
• Willingness to participate and provide written informed consent

#### 2.1.2. Exclusion Criteria

• Previous CABG surgery
• Left ventricular ejection fraction < 30% or decompensated heart failure
• History of aortic valve replacement or ascending aortic surgery
• Severe aortic stenosis or advanced aortic regurgitation (or indication for simultaneous valve surgery)
• Bicuspid aortic valve
• Ascending aortic aneurysm > 4.5 cm
• Active cancer
• Pregnancy
• Regular hemodialysis program

These criteria ensured that the study population was homogeneous and appropriate for evaluating the association between aortic cellular senescence and CAD complexity by excluding conditions that could potentially affect the expression of senescence‐associated proteins analyzed in our study.

**Figure 1:**
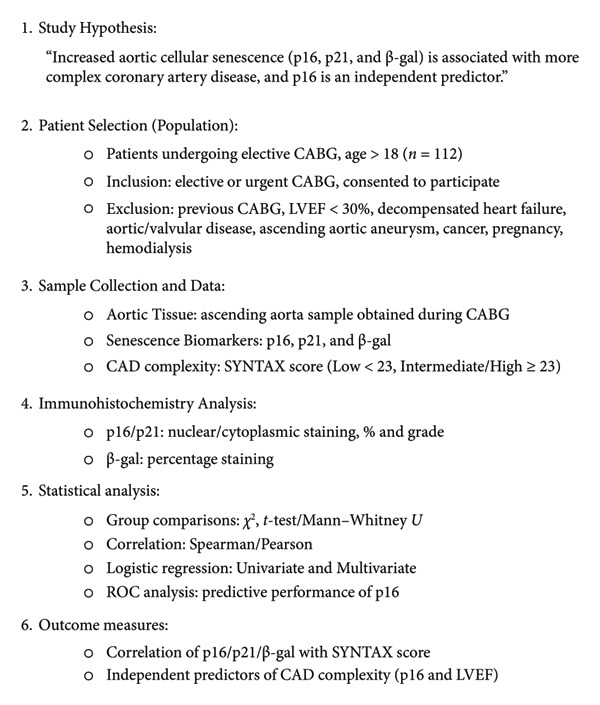
Study design flowchart. CAD, coronary artery disease; CABG, coronary artery bypass grafting; LV EF, left ventricular ejection fraction.

A study flowchart including patient selection, assessment of cellular senescence by immunohistochemical method and complexity of CAD by SYNTAX score, and statistical analysis is described in Figure 1.

The aortic tissue that was removed from the ascending aorta during the CABG operation was preserved in 10% formaldehyde solution. The aortic tissue samples were fixed in the solution for a minimum of 6 h and a maximum of 48 h. After fixation, paraffin blocks were created for each sample. One section from each block was taken for hematoxylin and eosin staining, and three sections were taken on positively charged slides for immunohistochemical study. The immunostaining protocol for p16, p21, and β‐gal was performed using positive control tissues on a fully automated device (Ventana). Four‐micron sections were taken from formalin‐fixed, paraffin‐embedded blocks. After some routine processes, the sections were kept in endogenous peroxide block for 5 min, and p16 (clone: IHC016‐0.1, monoclonal mouse Anti‐Human INK4A, GenomeMe, 1/100 dilution [1:50–120 dilution range]), p21 (clone: WAF1 DCS‐60.2 Cell Marque, 1/100 dilution [1:50–200 dilution range]), and β‐gal ([B‐12] Conc. 1 mL 1/100 dilution [1:50–500 dilution range]) oncoproteins were applied separately for 30 min for each one. Immunohistochemical staining was performed on the VENTANA BenchMark ULTRA instrument as described by the manufacturer’s protocol.

**Figure 2:**
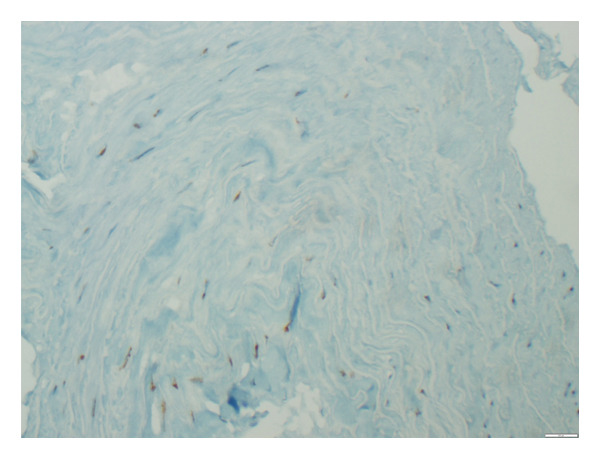
β‐gal staining in the aortic tissue showing 10% positivity (grade 1), documented at × 100 magnification.

**Figure 3:**
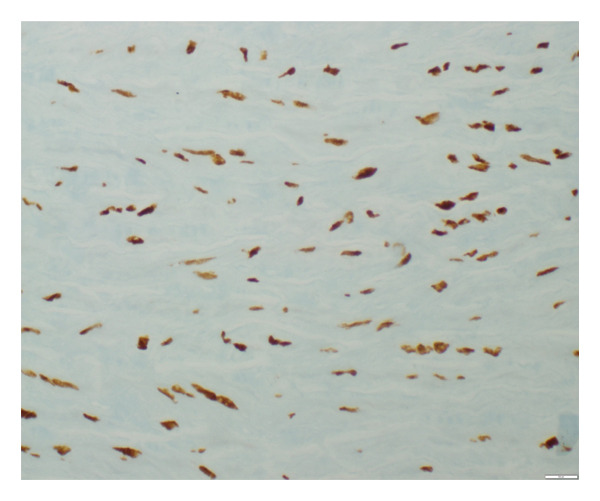
β‐gal staining in the aortic tissue showing 70% positivity (grade 2) documented at × 400 magnification level.

**Figure 4:**
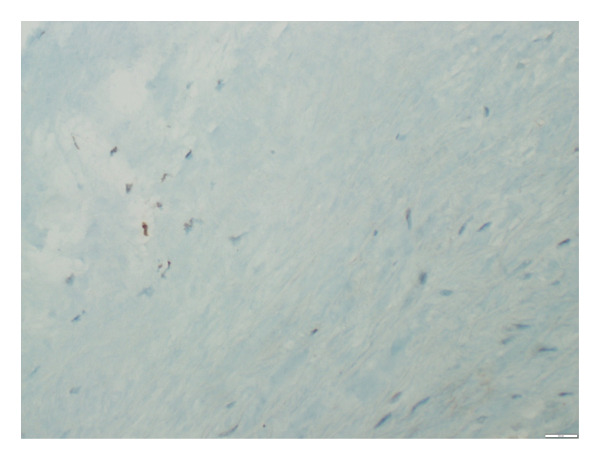
p16 staining in the aortic tissue showing 5% positivity (grade 1) documented at × 100 magnification level.

**Figure 5:**
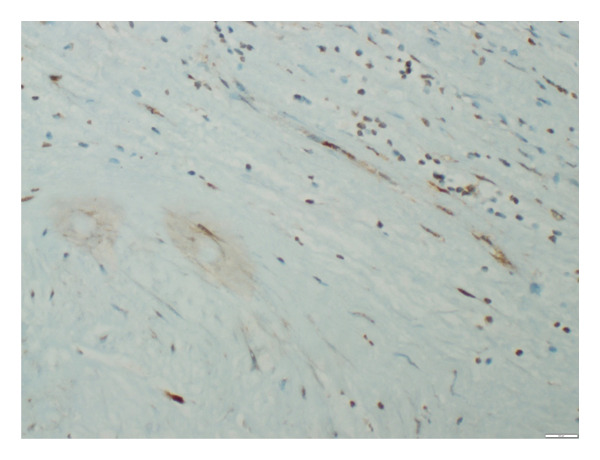
p16 staining in the aortic tissue showing 50% positivity (grade 2) documented at × 100 magnification level.

**Figure 6:**
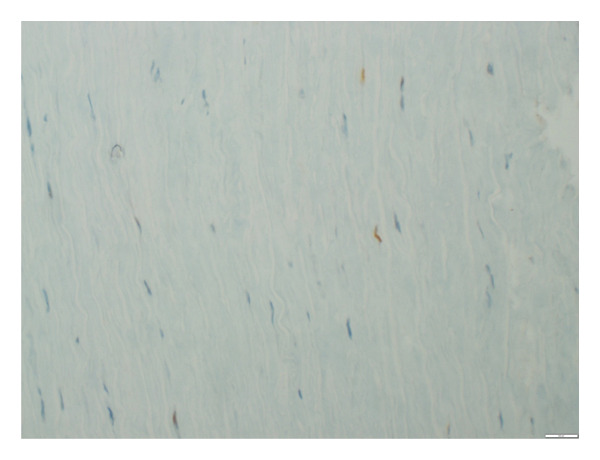
p21 staining in the aortic tissue showing 5% positivity (grade 1) documented at × 100 magnification level.

**Figure 7:**
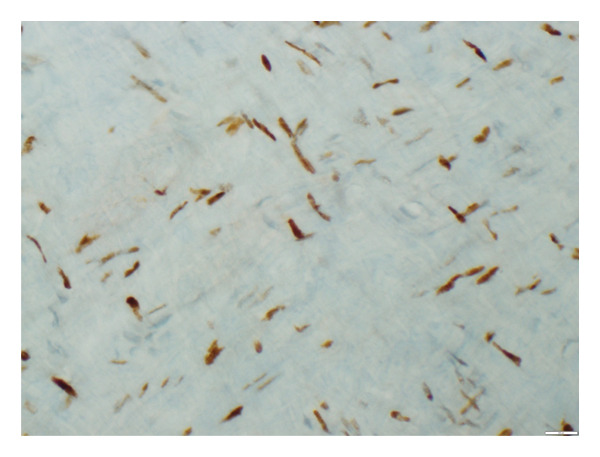
p21 staining in the aortic tissue showing 50% positivity (grade 2) documented at × 400 magnification level.

The stained tissue sections were evaluated independently by two experienced pathologists who were blinded to the clinical data or the SYNTAX scores of the patients. Nuclear staining and cytoplasmic staining were analyzed for p16 and β‐Gal, respectively. For p21, both cytoplasm and nuclear staining were analyzed. Counting of biomarker‐positive cells was performed under a light microscope. All tissue sections were scanned to estimate the average percentage of staining at high magnification field (X400). Both p16 and p21 were evaluated in 3 categories as negative staining, weak staining, and strong staining. In addition, the percentage of direct staining was given for β‐Gal, p16, and p21 staining (Figures 2, 3, 4, 5, 6, 7). Similarly, both p16 and p21 staining were graded as 0% negative (grade 0), 0%–10% weakly positive (grade 1), and > 10%–100% positive (grade 2). Evaluation of β‐gal staining was only given as the percentage of direct staining.

The severity and extent of CAD of the patients were calculated using SS, which is a scoring system helped to prefer whether percutaneous or surgical revascularization strategies. In our study, SS was calculated by two experienced blinded interventional cardiologists who were unaware of the clinical and laboratory results of the patients. First, significant CAD was defined as ≥ 50% stenosis in any epicardial coronary artery with a vessel diameter of ≥ 1.5 mm. Then, SS was calculated using the SS calculator (https://www.syntaxscore.com), and cut‐off values were determined as previously described in the SYNTAX study (low: ≤ 22, intermediate: 23–32, and high: ≥ 33). Our patients were categorized into two groups with low SS (< 23) and intermediate and high SS (≥ 23), respectively [21, 22].

### 2.2. Statistics

Categorical variables are given as frequency and percentage. The chi‐square (*χ* ^2^) test was used to compare categorical variables between groups. Continuous variables are presented as mean ± standard deviation (if normal distribution) and median (if not normal distribution). The Kolmogorov–Smirnov test was used to evaluate whether the variables were normally distributed. Student’s *t*‐test or Mann–Whitney *U* test was used to compare continuous variables between groups depending on whether they were normally distributed or not. Univariate and multivariate logistic regression analyses were performed to determine independent factors of CAD severity. Only variables with a *p* value of less than 0.05 in the univariate analysis were included in the multivariate logistic regression analysis. Variables that correlated well with each other were not included in the same analysis but were handled separately. Receiver operating characteristic (ROC) curve analysis was used to evaluate the predictive performance, sensitivity, specificity, and cut‐off value of p16 staining percentage for predicting CAD severity. Results were evaluated with a 95% confidence interval (CI) and a significance level of *p* < 0.05. All statistical analyses were performed using the Statistical Package for the Social Sciences Version 24.0 (IBM Corp., Armonk, NY, USA).

## 3. Results

The mean age of study patients (*n* = 112) was 60.6 ± 8.7 (73.2% male and 26.8% female). The patients were divided into 2 groups according to complexity of CAD as low SS (< 23) and intermediate and high SS (≥ 23). The mean SS of the low SS group was 17.1 ± 3.6, while the mean SS of the intermediate and high SS group was 28.5 ± 5.2. The distribution of the number of coronary lesions in different coronary arteries was similar in two study groups (Table 1).

**Table 1:** Coronary angiographic findings and severity of coronary artery disease in the study population.

Variables	All patients (*n* = 112)	Low SYNTAX score (SS) (*n* = 65, 58%)	Intermediate‐high SYNTAX score (SS) (*n* = 47, 42%)	*p* value
LMCA, *n* (%)	29 (26.1)	17 (26.6)	12 (25.5)	0.903
LAD, *n* (%)	104 (93.7)	58 (90.6)	46 (97.9)	0.121
Cx, *n* (%)	94 (84.7)	56 (87.5)	38 (80.9)	0.337
RCA, *n* (%)	87 (78.4)	48 (75.0)	39 (83.0)	0.313
SYNTAX score	21.9 ± 7.1	17.1 ± 3.6	28.5 ± 5.2	**< 0.001**

*Note:* Cx, circumflex artery; LAD, left anterior descending artery. Bold values indicate *p* < 0.05 as statistically significant.

Abbreviations: LMCA, left main coronary artery; RCA, right coronary artery.

No significant difference was found between the study groups in terms of demographic characteristics, clinical presentation, and medical and surgical treatments except the left ventricular ejection fraction which was significantly lower (49.9 ± 8.5 vs. 53.7 ± 7.0; *p* = 0.01) in the intermediate and high SS group. All clinical and demographic characteristics of the patients are shown in Table 2.

**Table 2:** Basic demographic and clinical characteristics of the study group.

Variables	All patients (*n* = 112)	^‡^Low SYNTAX score (SS) (*n* = 65, 58%)	İntermediate‐high SYNTAX score (SS) (*n* = 47, 42%)	*p* value
Male gender, *n* (%)	82 (73.2)	49 (75.4)	33 (70.2)	0.542
Age (Years)	60.6 ± 8.7	59.3 ± 8.6	62.5 ± 8.6	0.058
Hypertension, *n* (%)	55 (49.1)	33 (50.8)	22 (46.8)	0.679
Diabetes mellitus, *n* (%)	61 (54.5)	37 (56.9)	24 (51.1)	0.539
Hyperlipidemia, *n* (%)	51 (45.5)	32 (49.2)	19 (40.4)	0.356
Family history, *n* (%)	26 (23.2)	15 (23.1)	11 (23.4)	0.968
Cigarettes, *n* (%)	48 (42.9)	27 (41.5)	21 (44.7)	0.740
CAD (old diagnosis), *n* (%)	37 (33)	24 (36.9)	13 (28.3)	0.340
Peripheral artery disease, *n* (%)	12 (10.7)	6 (9.2)	6 (12.8)	0.551
CHF, *n* (%)	9 (8.0)	3 (4.6)	6 (12.8)	0.117
COPD, *n* (%)	4 (3.6)	2 (3.1)	2 (4.3)	0.740
CKD, *n* (%)	2 (1.8)	0 (0.0)	2 (4.3)	0.093
SVO, *n* (%)	11 (9.8)	5 (7.7)	6 (12.8)	0.373
ASA, *n* (%)	58 (51.8)	35 (53.8)	23 (48.9)	0.608
Dual antiplatelet, *n* (%)	13 (11.6)	7 (10.8)	6 (12.8)	0.745
Oral anticoagulant, *n* (%)	1 (0.9)	0 (0)	1 (2.1)	0.237
ACEI/ARB	46 (41.1)	30 (46.2)	16 (34)	0.199
Beta‐blocker, *n* (%)	41 (36.6)	24 (36.9)	17 (36.2)	0.935
KKB, *n* (%)	13 (11.6)	10 (5.4)	3 (6.4)	0.142
Statin, *n* (%)	37 (33)	23 (35.4)	14 (29.8)	0.534
Acute coronary syndrome, *n* (%)	55 (49.1%)	28 (43.1%)	27 (57.4)	0.133
LVEF, *n* (%)	52.1 ± 7.9	53.7 ± 7.0	49.9 ± 8.5	**0.010**
Retrograde approach, *n* (%)	38 (33.9)	21 (32.3)	17 (36.2)	0.313
Postop AF, *n* (%)	13 (11.6)	6 (9.2)	7 (14.9)	0.356
Mortality (1 year), *n* (%)	11 (9.8)	1 (1.5)	10 (21.3)	**0.001**
MACE, *n* (%)	22 (19.6)	2 (3.1)	20 (42.6)	**< 0.001**
Intensive care stay (days), median, [IQR]	4.0 [3.0–5.0]	4.0 [3.0–5.0]	4.0 [3.0–6.0]	0.107
Follow‐up time (total days), median, [IQR]	355.0 [292.3–424.0]	367.0 [301.5–424.0]	335.0 [276.0–425.0]	**0.022**

*Note:* ASA, acetylsalicylic acid; CKD, chronic renal failure; CKB, calcium channel blocker; CVO, cerebrovascular. Bold values indicate *p* < 0.05 as statistically significant.

Abbreviations: ACEİ, angiotensin‐converting enzyme inhibitor; AF, atrial fibrillation; ARB, angiotensin receptor blocker; CAD, coronary artery disease; CHF, congestive heart failure; COPD, chronic obstructive pulmonary disease; LVEF, left ventricular ejection fraction; MACE, major adverse cardiac event.

^‡^Low SS < 23; intermediate‐high SS ≥ 23.

The major adverse cardiovascular events documented during the follow‐up period were found to be significantly higher in the intermediate and high SS group (*p* < 0.001). One‐year mortality during follow‐up was significantly higher in the intermediate and high SS group (*p* = 0.001). The total follow‐up period was statistically significantly higher in the low SS group (*p* = 0.022), indicating better survival. No statistically significant difference was found between the two groups in terms of postoperative development of atrial fibrillation.

There has been no significant difference in the laboratory parameters of two study groups except 3 parameters: (1) Postoperative platelets (191 ± 59 × 10^3^ vs. 170 ± 55 × 10^3^) were significantly low in patients with intermediate and high SS (*p* = 0.049); (2) postoperative troponin *T* value (380 [285–594] vs. 476 [313–1000] pg/mL); and (3) C‐reactive protein levels (3.6 [1.7–8.9] vs. 6.1 [2.4–18.0] mg/L) were both significantly higher in the intermediate and high SS group. Detailed hematological and biochemical parameters of the study groups are shown in Table 3.

**Table 3:** Hematological and biochemical parameters of the study group.

Variables	All patients (*n* = 112)	Low SYNTAX score (SS) (*n* = 65, 58%)	İntermediate‐high SYNTAX score (SS) (*n* = 47, 42%)	*p* value
Fasting blood glucose, mg/dL, median, [IQR]	126.5 [101.0–158.5]	126.0 [101.0–161.5]	127.0 [99.0–157.0]	0.528
Creatinine, mg/dL, median, [IQR]	0.82 [0.72–0.96]	0.81 [0.72–0.90]	0.82 [0.73–1.04]	0.174
Total cholesterol (mg/dL)	185.9 ± 52.6	189.5 ± 52.6	180.9 ± 52.7	0.401
LDL‐C (mg/dL)	112.9 ± 45.2	117.4 ± 46.5	106.7 ± 43.2	0.217
HDL‐C (mg/dL)	40.4 ± 10.4	41.7 ± 11.1	38.6 ± 9.1	0.116
TG, mg/dL, median, [IQR]	182.5 [147.0–214.0]	152.0 [101.5–190.0]	156.0 [124.0–217.0]	0.323
Uric acid, mg/dL, median, [IQR]	4.3 [3.5–5.5]	4.2 [3.5–5.2]	4.8 [3.5–6.1]	0.074
Albumin (g/dL)	4.1 ± 0.44	4.2 ± 0.47	4.1 ± 0.41	0.889
Preop troponin *T* (pg/mL)	7.0 ± 0.68	7.0 ± 0.65	7.0 ± 0.73	0.991
CRP, mg/L, median, [IQR]	4.3 [2.0–11.9]	3.6 [1.7–8.9]	6.1 [2.4–18.0]	**0.028**
Preop WBC (10^3^ μL)	8.4 ± 2.3	8.4 ± 2.2	8.3 ± 2.4	0.181
Neutrophil (10^3^ μL)	5.6 ± 2.0	5.8 ± 1.8	5.5 ± 2.2	0.301
Lymphocyte (10^3^ μL)	2.1 ± 0.6	2.1 ± 0.7	2.0 ± 0.9	0.915
Preop PLT (10^3^ μL)	241 ± 82	237 ± 68	247 ± 98	0.515
Preop hemoglobin (g/L)	13.5 ± 2.0	13.6 ± 1.2	13.2 ± 2.3	0.201
RDW (%)	13.7 ± 1.2	13.8 ± 1.2	13.6 ± 1.1	0.316
Postop hemoglobin (g/dL)	9.4 ± 1.8	9.4 ± 1.9	9.3 ± 1.7	0.704
Postop WBC (10^3^ μL)	11.4 ± 3.4	11.8 ± 3.6	10.8 ± 3.2	0.130
Postop PLT (10^3^ μL)	182 ± 58	191 ± 59	170 ± 55	**0.049**
Postop creatinine, mg/dL, median, [IQR]	0.90 [0.80–1.20]	0.90 [0.70–1.10]	0.90 [0.80–1.20]	0.087
Postop troponin *T* (pg/mL)	416 [289–701]	380 [285–594]	476 [313–1000]	**0.007**

*Note:* PLT: platelet, TG: triglyceride. Bold values indicate *p* < 0.05 as statistically significant.

Abbreviations: CRP, C‐reactive protein; HDL‐C, high‐density lipoprotein cholesterol; LDL‐C, low‐density lipoprotein cholesterol.

In case of senescence proteins, a mildly significant but negative correlation was found between age and percentage of β‐gal expression in the aortic tissue (*r* = −0.217, (*p* = 0.022). Beyond that, the percentage of β‐gal‐positive senescence cells was similar in the study groups. There was no correlation between age and expression of p16 and p21 in terms of percentile (*r* = 0.098 and *p* = 0.302; *r* = −0.096 and *p* = 0.314, respectively) and grade (*r* = 0.114 and *p* = 0.229; *r* = 0.068 and *p* = 0.478, respectively). However, percentage and grade of both p16‐ and p21‐positive senescent cells in the aortic tissue were found to be significantly higher in the intermediate and high SS group (Table 4).

**Table 4:** Immunohistochemical parameters of the study group.

Variables	All patients (*n* = 112)	Low SYNTAX score (SS) (*n* = 65, 58%)	İntermediate‐high SYNTAX score (SS) (*n* = 47, 42%)	*p* value
β‐gal. (%)	59.4 ± 15.6	58.3 ± 15.6	60.9 ± 15.7	0.371
p16, (%), median, [IQR]	10.0 [1.0–40.0]	5.0 [1.0–40.0]	15.0 [5.0–60.0]	**0.015**
p16 grade median, [IQR]	1.0 [1.0–2.0]	1.0 [0.0–2.0]	1.0 [1.0–2.0]	**0.035**
p21, (%), median, [IQR]	13.3 [5.9–39.2]	10.0 [5.0–30.0]	23.3 [8.3–46.7]	**0.015**
p21 grade median, [IQR]	1.0 [1.0–2.0]	1.0 [1.0–2.0]	2.0 [1.0–2.0]	**0.03**

*Note:* Bold values indicate *p* < 0.05 as statistically significant.

In univariate logistic regression analysis, left ventricular ejection fraction (OR, 0.938 [95% CI, 0.892–0.987]; *p* = 0.013), C‐reactive protein (OR, 1.036 [95% CI, 1.002–0.1.072]; *p* = 0.040), p21 percentage (OR, 1.025 [95% CI, 1.004–1.047]; *p* = 0.017), p16 percentage (OR, 1.017 [95% CI, 1.003–1.032]; *p* = 0.018), p21 grade (OR, 1.937 [95% CI, 1.055–3.555]; *p* = 0.033), and p16 grade (OR, 1.740 [95% CI, 1.033–2.932]; *p* = 0.037) were found to be associated with intermediate and high SS group (Table 5). These data were analyzed in the multivariate logistic regression analysis. As p21 and p16 were significantly correlated with each other, percentage and grade evaluation were evaluated in two separate regression analysis models (model 1 and model 2). β‐gal was not included in this analysis as it did not differ between the groups. In model 1, left ventricular ejection fraction (OR, 0.942 [95% CI, 0.892–0.995]; *p* = 0.031) and p16 expression percentage (OR, 1.016 [95% CI, 1.000–1.031]; *p* = 0.047) were found to be independent predictors of intermediate‐high SS group. In model 2, left ventricular ejection fraction (OR, 0.931 [95% CI, 0.881–0.984]. *p* = 0.012) was found to be an independent predictor of intermediate and high SS group.

**Table 5:** Factors found to be independently associated with coronary artery disease severity in univariate and multivariate logistic regression analysis.

Variables	Univariate OR (95% CI)	*p* value	Multivariate 1 OR (95% CI)	*p* value	Multivariate 2 OR (95% CI)	*p* value
LVEF	0.938 (0.892–0.987)	**0.013**	0.942 (0.892–0.995)	**0.031**	0.931 (0.881–0.984)	**0.012**
CRP	1.036 (1.002–1.072)	**0.04**	1.021 (0.984–1.059)	0.267	1.018 (0.982–1.056)	0.335
p21, (%)	1.025 (1.004–1.047)	**0.017**	1.019 (0.997–1.042)	0.090	—	—
p16, (%)	1.017 (1.003–1.032)	**0.018**	1.016 (1.000–1.031)	**0.047**	—	—
p21 grade	1.937 (1.055–3.555)	**0.033**	—	—	1.947 (0.972–3.900)	0.06
p16 grade	1.740 (1.033–2.932)	**0.037**	—	—	1.453 (0.819–2.580)	0.202

*Note:* LVEF, left ventricular ejection fraction separately. Bold values indicate *p* < 0.05 as statistically significant.

Abbreviation: CRP, C‐reactive protein.

**Figure 8:**
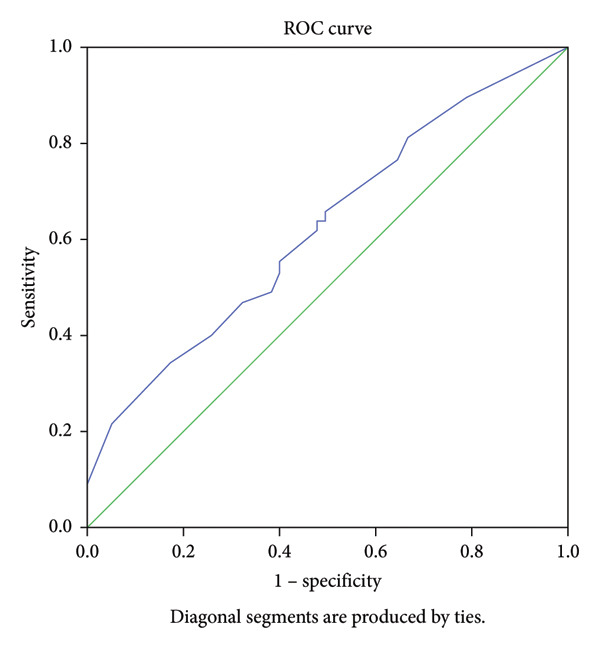
ROC curve showing the power of p16 value in predicting the severity of coronary artery disease. AUC 0.625 (95% CI: 0.519–0.730; *p* = 0.025). A p16 ratio > 5.5% cut‐off value indicates the severity of coronary artery disease with 66% sensitivity and 51% specificity. AUC: area under the curve; ROC: receiver operating characteristics.

ROC analysis was also performed to test the predictive performance of p16 and its sensitivity and specificity in predicting the complexity of CAD. The AUC value was 0.625 (95% CI = 0.519–0.730, *p* = 0.025), and it had the predictive power of the complexity of CAD with 66% sensitivity and 51% specificity for a cut‐off value > 5.5% (Figure 8).

## 4. Discussion

The combination of p16, p21, and ß‐gal was used as biomarkers of cellular senescence in our study. A negative correlation between chronological age and expression of β‐gal in the aortic tissue was interestingly found. Otherwise, our findings indicated that chronological age was not related to expressions of both p16 and p21 in the ascending aorta in our study. Our findings are compatible with previous studies which indicate that cellular senescence is not usually related to chronological age [7, 8]. The heterogeneity of increased biological age with respect to chronological age is believed to result from accelerated cellular senescence [23–25]. Nevertheless, the exact mechanisms by which the accumulation of senescent cells results in deviations in biological age as well as the development of cardiovascular diseases have not been fully elucidated. However, new evidences support that biological age could be improved by risk factor modification, such as management of high blood pressure, glycemic control and stress reduction, exercise, and smoking cessation, all of which are also crucial for preventing CAD [26–28].

The relationship between nonatherosclerotic heart diseases and accumulation of senescent cells in cardiovascular tissue was previously investigated in both animal and human studies [29–32]. Indeed, it is suggested that increased senescent cells in cardiovascular tissues could trigger or exacerbate the onset and progression of numerous cardiovascular diseases, including atherosclerosis, arterial stiffening, aortic aneurysms, myocardial fibrosis, and congestive heart failure [22, 33–35]. Furthermore, removal of p16‐positive senescent cells from plaques in an animal model suppressed pathological atherosclerotic changes in LDL receptor‐deficient mice [36, 37]. In addition, senolytic and senomorphic drugs have recently been investigated in human studies as promising and innovative therapies to prevent cardiovascular diseases [38–41]. Local administration of senolytics could also be used in the treatment of coronary atherosclerosis, such as sirolimus‐eluting stents. A senolytic‐eluting stent might similarly provide antiatherogenic benefits without much concern about off‐target effects [42]. However, there is still limited evidence about the role of senolytic or senomorphic therapies in the prevention and treatment of cardiovascular diseases.

In our study, patients with aortic aneurysm, bicuspid valve disease, and advanced aortic valvular disease were excluded because of evidences showing increased senescence proteins in patients with bicuspid aortic valve, aortic aneurysm, and aortic stenosis [9, 10]. The expression of p16 was found significantly higher in areas of moderate to high calcification and/or ossification and severe fibrosis compared to normal or mildly fibrotic and/or calcified valvular tissue in patients with severe aortic stenosis [10]. In this study, the expression of p16 was also higher in interstitial valve cells in proportion to the degree of fibrosis. Nevertheless, some cardiac and noncardiac diseases, whose effects on senescence biomarkers have not yet been fully investigated, might not have been excluded in our study patients.

In conclusion, our study is the first human study analyzing correlations between proportion of senescence protein expressions in the aortic tissue and the complexity of coronary artery stenoses. It has been shown that the higher expression of senescence proteins in the aortic tissue correlated with the more complex CAD in our study. The demonstration of significant relationships between increased p16 and p21 expressions in aortic tissue and complexity of CAD indirectly indicates a possible role of cellular senescence in the development of coronary atherosclerosis. A higher percentage of p16 expression in the aortic tissue was also found to be an independent predictor of more complex CAD. Our results would inspire further studies to suppress or eliminate senescent cells in the coronary arteries for the prevention and treatment of CAD.

## 5. Strengths and Limitations of the Study

This study has several strengths and limitations. It is the first to directly assess cellular senescence biomarkers in vascular tissue (ascending aorta) obtained from living individuals, providing unique biological insight from the target vascular structure rather than peripheral blood. The use of multiple senescence markers (p16, p21, and β‐gal) strengthens the validity of the findings, and the demonstrated association between their expression and the complexity of CAD represents a novel contribution to aging research. However, the cross‐sectional design limits causal interpretation, and longitudinal evaluation of vascular senescence remains ethically and practically unfeasible. The study population consisted of patients undergoing elective or urgent CABG, which may restrict generalizability, although inclusion of patients with low and intermediate SYNTAX scores allowed representation of a broad CAD spectrum. Coronary tissue samples could not be obtained for ethical reasons. Thus, aortic tissue was analyzed as a surrogate, which may not fully reflect coronary senescence processes. No a priori power analysis was performed, and potential confounding effects from unmeasured comorbidities cannot be entirely excluded. Functional assays and broader inflammatory profiling were also not performed. Finally, while p16 showed modest predictive power and β‐gal did not correlate with CAD complexity, these findings are consistent with prior reports on marker specificity. Future studies with larger, longitudinal cohorts and expanded biomarker panels are warranted to further clarify the role of vascular senescence in coronary atherosclerosis.

## Conflicts of Interest

The author declares no conflicts of interest.

## Funding

No funding was received for this manuscript.

## Data Availability

The data that support the findings of this study are available upon request from the corresponding author. The data are not publicly available due to privacy or ethical restrictions.
